# Factors Influencing Elderly Consumers’ Preferences for Edible Gels: Insights from Slovakia

**DOI:** 10.3390/gels10100610

**Published:** 2024-09-24

**Authors:** Melina Korčok, Miroslav Veverka, Kristina Nakonechna, Simona Škrípová, Vladimir Vietoris

**Affiliations:** 1 Institute of Food Sciences, Faculty of Biotechnology and Food Sciences, Slovak University of Agriculture in Nitra, Trieda A. Hlinku 2, 949 76 Nitra, Slovakia; simona1skripova@gmail.com (S.Š.); vladimir.vietoris@uniag.sk (V.V.); 2 Certex, a.s., Radlinského 9, 812 37 Bratislava, Slovakia; miroveve@gmail.com; 3 Department of Food Analysis and Nutrition, University of Chemistry and Technology, Technická 5, 166 28 Prague, Czech Republic; nakoneck@vscht.cz

**Keywords:** edible gels, consumer preferences, seniors, fiber, beta-glucan, arabinogalactan

## Abstract

As dietary needs shift with the growing and aging population, there is a demand for food products that meet nutritional, safety, and tribological requirements while being cost-effective. Seniors must be given significant consideration in new product development. This study examines consumer preferences for arabinogalactan (AG) and beta-glucan (BG) hydrogels with vanilla and coffee-biscuit flavors, using consumer tests (*N* = 80) and an online survey (*N* = 852). It focuses on the gels’ physical properties, such as texture and viscosity, and their impact on sensory perception. The use of two different gel-forming polysaccharides, each with a unique sensory profile, was observed to affect the sensory properties of the resulting gels and subsequently influence product acceptance. This study analyzed preferences across three age groups: young (18–39 years), middle-aged (40–59 years), and older adults (60+ years). The results showed that seniors preferred AG-based gels. Significant attributes such as the intensity of flavor and bitter taste influenced the overall liking of the gels. Texture also notably impacted preferences. The survey findings revealed statistically significant (*p* < 0.05) differences in preferences between older adults and younger age groups. Tailoring product development and marketing strategies based on age and sensory preferences could enhance consumer acceptance of edible gels.

## 1. Introduction

Given the various health requirements and the specific nutritional needs of the population, it is necessary to develop food products capable of meeting them [1]. The growing world population, along with changing dietary behaviors, preferences, and eating behaviors, are leading to the need for new food products with certain requirements in terms of nutritional composition, safety, and environmental impact. In response to this challenge, food scientists have turned their attention to gels based on food biopolymers as a promising solution [2,3,4]. In particular, protein and polysaccharide-based gels have received significant interest among food scientists due to their biocompatibility, biodegradability, nutritional properties, and palatability [2,5,6].

Polysaccharide-based gels offer a wealth of advantages, ranging from their natural origin to their functional properties. Among these, fibers play a crucial role in enhancing nutritional value and promoting overall health. Fiber intake has many health benefits [7], including the regulation of blood sugar levels and cholesterol levels and improved gut health. Additionally, it has been associated with improved cognitive function and a reduced risk of cognitive decline in older adults [8,9,10,11]. Studies [12,13] also suggest that adequate fiber intake may reduce the risk of coronary heart disease and various types of cancer, particularly colon cancer. This adaptability makes them not only a dietary solution but also a medium for delivering therapeutic benefits, further enhancing their value in senior care [5,14,15].

Fibers such as beta-glucan (BG) are found naturally in certain foods, particularly oats, barley, and some types of mushrooms like *Pleurotus ostreatus*, commonly known as oyster mushrooms. BG is renowned for its various health benefits, particularly in supporting heart health [7] and boosting the immune system [13]. It has also been shown to improve glycemic control, making it beneficial for managing diabetes. Similarly, arabinogalactan (AG), found in the cell walls of many plants, including *Larix sibirica*, is recognized for its prebiotic properties [16] and immune-modulating effects [17]. AG holds promise in promoting gut health [18] and overall well-being in aging individuals. It supports the growth of beneficial gut bacteria, enhances immune function, and has been linked to improved digestive health [17,19].

In addition to their nutritional benefits, their sensory properties, especially taste, texture, appearance, and aroma, are crucial for their acceptance and consumption, especially by seniors. As people age, they often experience changes that directly or indirectly affect the food intake process, such as reduced saliva production, dental problems, and weakened swallowing mechanisms [5,14,20]. Various other changes occur throughout the digestive system. Seniors often face challenges with diminished senses of taste and smell, poor appetite, thirst dysregulation, and decreased sensory perception, making it vital for foods to be optimal. Consequently, these changes can lead to difficulties in food consumption, increasing the risk of malnutrition [21,22] and various health problems. Therefore, it is essential to provide appropriate food that is not only nutritious but also sensory-suitable, easy to chew, swallow, and enjoy.

Polysaccharide-based gels represent food gels intended for direct consumption and offer an ideal and effective solution for seniors due to their suitable texture, which requires minimal effort to chew, and their desirable sensory properties and potential for incorporating biologically active substances and flavoring agents [20]. Determining the key physical and sensory attributes that appeal to consumers is crucial to achieving market success with edible gels. Understanding and optimizing these characteristics ensures that the product meets the preferences and expectations of the target demographic, thereby increasing the likelihood of its adoption and success in the marketplace. Sensory and consumer sciences provide valuable tools to understand better products and consumer preferences that can help reduce the risk of product failure. A widely accepted sensory method for measuring consumer preferences is the acceptability test, which often uses a nine-point hedonic scale [23].

The objective of this study is to investigate consumer preferences regarding edible gels containing AG and BG through consumer tests (*N* = 80) and online surveys (*N* = 852). Insights from a defined sample of respondents were explored in order to understand the acceptability and specific preferences regarding AG and BG gels. The online questionnaire aimed to investigate the preferences of three age categories (young adults, middle-aged adults, and older adults) with the main focus of exploring the hypothesis that age as a demographic factor influences consumer preferences regarding the characteristics and requirements of edible gels. In addition, the physical properties of the gels, such as texture and viscosity, were analyzed and their influence on the perception of sensory properties was investigated. The aim was to identify key attributes influencing consumer acceptance of these gels, determine whether age influences edible gel preferences, and suggest strategies for the development of gel products.

## 2. Results and Discussion

### 2.1. Viscosity and Texture

The viscosity and texture parameters of the evaluated samples were compared using Duncan ANOVA and REGWQ tests, and the results are shown in Table 1. A *p*-value of <0.0001 was found for all parameters, indicating that the differences between the samples are statistically significant for all evaluated properties. The highest values for viscosity, firmness, and consistency and the lowest for cohesiveness and work of cohesion were observed in the BG-based gel with coffee-biscuit flavor indicating a stronger and less cohesive structure of this gel. On the contrary the AG-based gel with vanilla flavor was characterized by the lowest viscosity, firmness, and consistency values and the highest values for cohesiveness and work of cohesion. The observed differences in gel properties can be attributed to the different molecular structures and interactions with water exhibited by the two different types of polysaccharides [24,25]. Yeast-extracted BGs exhibit lower solution viscosity compared to gums and lower gel-forming ability, as yeast-derived beta-glucans themselves form mechanically weak hydrogels [24]. In addition, statistically significant differences were also observed between the properties of samples manufactured from the same polysaccharides (e.g., BG or AG). We assume that BG, unlike soluble AG, forms a weak gel and appears less sensitive to the presence of additives.

### 2.2. Consumer Test

**Figure 1:**
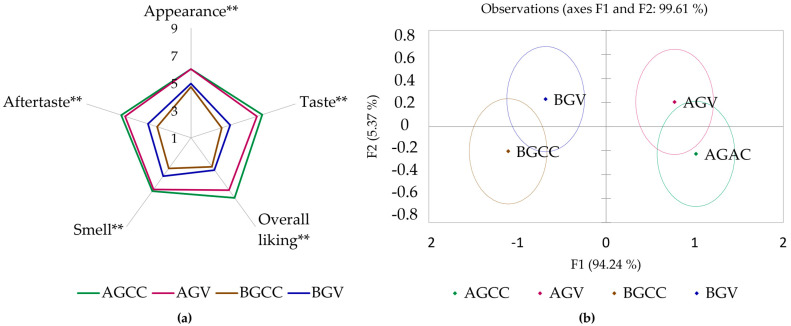
Results of sensory analysis of edible gels: (**a**) evaluation of sensory properties on a nine-point hedonic scale; (**b**) gel discrimination using linear discriminant analysis (LDA). Note: “**” indicates statistical significance at *p* < 0.05.

All attributes were found to differ significantly (*p* < 0.05) between samples (see Figure 1). Consumers predominantly preferred AG-based gels, with AGCC receiving consistently the highest scores across all attributes, indicating that it was the most preferred gel in terms of appearance, aroma, taste, aftertaste, and overall liking. The second most preferred gel was the vanilla-flavored variant of the AG-based gel (AGV), but it received slightly lower scores in some attributes compared to AGCC. BG gels, specifically BGV and BGCC, were awarded lower scores for most attributes, indicating a lower preference compared to the AG gels. According to the Wilks’ Lambda test (Rao’s approximation), there is a significant difference between groups (see Figure 1b). An overlay was observed between BG gels and simultaneously between AG-based gels, and it emphasizes the similarity of samples in AG and BG groups. This overlap within the groups highlights that the samples in the AG group are closely related in terms of their sensory properties, as are the samples in the BG group. These findings confirm that respondents had distinct preferences for gels depending on the type of polysaccharide used (AG or BG), demonstrating the different consumer perceptions between the two types of gels. Although the gels were rated on a nine-point scale, no gel was evaluated with a mean score higher than six, indicating a relatively low level of consumer preference for the current formulations. This points to the need for further optimization.

**Figure 2:**
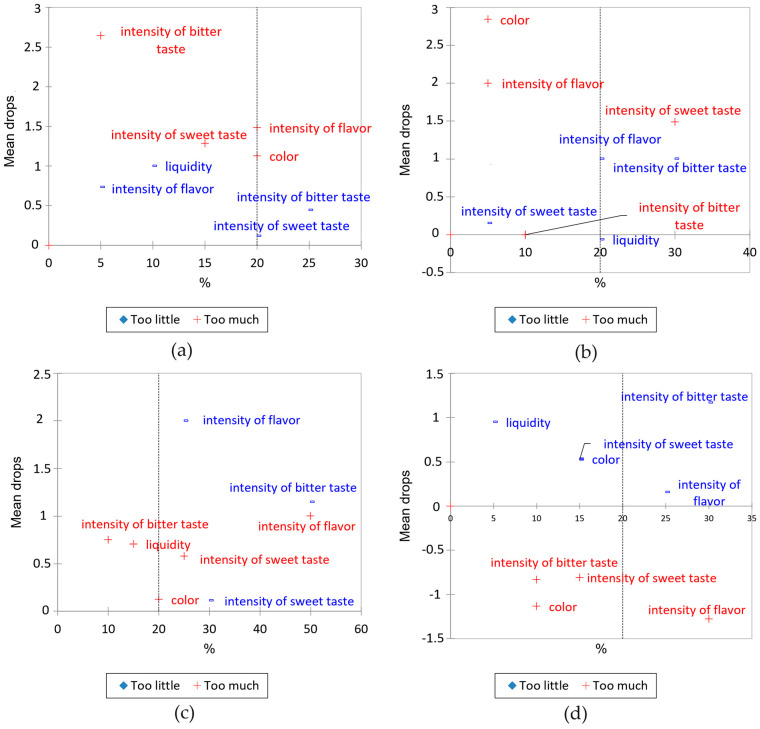
Mean drop plots for sensory attributes evaluated on JAR scale for samples: (**a**) AGCC–arabinogalactan gel with coffee-biscuit flavor; (**b**) AGV–arabinogalactan gel with vanilla flavor; (**c**) BGCC–beta-glucan gel with coffee-biscuit flavor; (**d**) BGV–beta-glucan gel with vanilla flavor.

The JAR scale was used to identify the specific attributes that most significantly influence consumer acceptance, followed by a penalty analysis conducted to identify key areas that require improvement. A penalty analysis is a graphical method used for identifying and quantifying the extent to which certain sensory attributes or flaws affect the overall perception and acceptability of a product. The sensory attributes that most significantly reduced the overall consumer liking of the product are shown in the top right quadrant of the graph. They represent the main areas that require improvement and optimization in order to increase overall consumer acceptance of the product [26,27]. The results are presented in Figure 2. AGCC was penalized for the low intensity of bitter taste, as 25% of consumers said that the gel was not bitter enough. About 20% of the evaluators were not satisfied with the intensity of the sweet taste attribute and considered it to be not sweet enough. At the same time, 20% of the evaluators described the color as “too much” as well as the intensity of the flavor. For the vanilla-flavored AG-gel, 30% of evaluators found the sweetness to be overpowering and the bitterness to be insufficient. Additionally, 20% of evaluators perceived the flavor intensity as “too little”. BG is often added to products in order to achieve a more nutritionally balanced product with health benefits; however, many studies have documented that consumer acceptability ratings have decreased following the addition of a higher concentration of this polysaccharide [28]. The coffee-biscuit flavored variant of BG gel was found to have the highest number of penalties—i.e., properties that adversely affected overall consumer preference. These findings are also consistent with the results of the evaluation of the gels using a nine-point hedonic scale, where this gel obtained the lowest score among all the samples. More specifically in the case of this sample, 50% of the evaluators perceived the intensity of the flavor as “too much”, and the same number of evaluators stated that the gel was “too little” bitter. The intensity of the sweet taste created the greatest discrepancy in the evaluators’ opinions, as 25% of the evaluators considered the gel too sweet and 30% considered it not sweet enough. The vanilla-flavored BG gel was penalized for insufficient flavor intensity, as 25% of the evaluators said the intensity was “too little”. Another penalty was for too little bitterness, as 30% of the evaluators indicated that the intensity of the bitter taste was “too little”. Previous research suggests that the addition of vanilla flavoring in conjunction with non-caloric sweeteners can increase the perceived sweetness of food products [29,30].

Interestingly, the gels with vanilla flavor were not perceived as not sweet enough, while the AG gel was penalized for having “too much” intensity of sweet taste. Age-related olfactory and gustatory changes are thought to lead to an overall reduction in flavor perception, resulting in foods appearing less intense or flavorful. Strategies recommended to compensate for this decline when developing foods for the elderly are the use of flavor enhancement/enrichment and the addition of irritants [14]. The penalty analysis in all plots confirmed the importance of harmonizing sensory attributes to increase overall consumer acceptability. In the case of the AG gel with coffee-biscuit, only one penalty, “too little” intensity of bitter taste, exceeded the 20% threshold. In addition, this gel scored highest on the nine-point hedonic scale, indicating that it has the highest negative impact on the overall quality of the product. 

### 2.3. Correlation between Physical Properties and Sensory Evaluation

**Figure 3:**
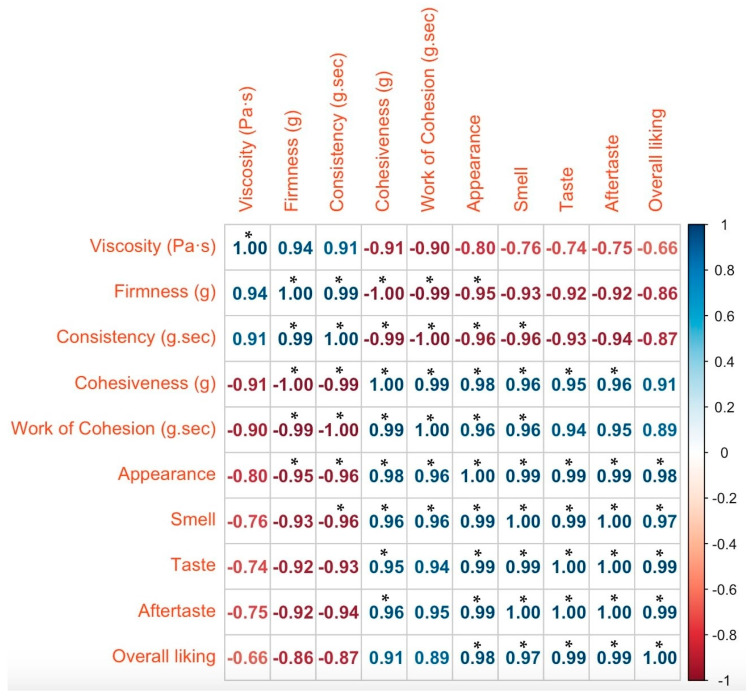
Pearson’s correlation between analyzed physical parameters and sensory attributes. Note: “*” indicates where the correlation is significant (*p* < 0.05).

The relationship between the physical properties of the gels and the sensory attributes was investigated, and the results are shown in Figure 3. Sensory attributes such as appearance, aftertaste, taste, and smell were strongly positively correlated with each other, indicating that improving one attribute is likely to improve the others. The most significant correlations were found between taste and aftertaste (1.00), appearance and aftertaste (0.99), aftertaste and smell (1.00), and taste and smell (0.99). The appearance had a strong impact on the overall liking (0.98) of the gels, indicating that the appearance of the new product influences the overall acceptability of consumers [31,32]. Improvements in sensory attributes that are positively associated with overall liking could increase consumer satisfaction.

An analysis of correlations between sensory properties and physical data revealed several interesting relationships. Statistically significant negative relationships were confirmed for firmness and appearance (−0.95). The very strong negative correlation indicates that firmer gels are perceived as having a poorer appearance. In the study by Akesowan (2021) [33], a relationship between firmness and appearance of konjac jelly was observed, with samples with higher firmness values obtaining higher scores in the sensory evaluation of appearance. Hydrogels, which have different structural properties than traditional gels, were evaluated in this study, explaining the different relationships between the findings when comparing the sensory attributes of appearance and gel firmness. Baroyi et al. (2022) found that consumers tend to prefer softer gel textures, as evidenced by the lower hardness scores reported in the study [34]. A similar trend where an increase in the hardness of hydrogels led to a decrease in liking scores has been confirmed in several studies [35,36], and it is suggested that individual characteristics, such as gender and age, can play an important role in hardness perception [36]. 

Cohesiveness showed a consistent positive statistically significant correlation with sensory attributes, such as appearance, taste, aftertaste, and smell (for all *p* < 0.05), and not significant with overall liking (*p* = 0.125). This parameter reflects the stickiness or adhesive quality of the sample, as indicated by the maximum opposing force encountered during the probe’s retraction. The more negative the value, the greater the “sticky” or “cohesive” nature of the sample [37]. In our study, better scores for sensory attributes were given to the AG gel samples, which were less adhesive than the BG gel samples. In addition, some negative relationships were also observed, such as a strong negative correlation between consistency and work of cohesion (−1.00), cohesiveness (−0.99), appearance (−0.96), and taste (−0.93). Product consistency appears to be negatively related to most properties except for firmness (0.99) and viscosity (0.91), with which it correlates positively. Higher consistency indicates higher resistance of the gel to deformation or flow [37]. These correlations suggest that changes in consistency may have a significant impact on the sensory attributes and overall likeability. Gels with higher viscosity have advantages in terms of ease of swallowing, taste retention, and product stability. Swallowing safety also reduces the risk of aspiration or choking [38]. In our study, negative correlations between sensory properties and viscosity were observed but were not significant at the α = 0.05 level. A strong positive correlation was observed between viscosity and consistency (0.91) and firmness (0.94), demonstrating the relationship that an increase in gel viscosity would increase the two textural properties. On the contrary negative correlations were observed between viscosity and cohesiveness (−0.91) and work of cohesion (−0.90). As the work of cohesion represents the ability of the gel to retain its structure and resist deformation during testing [37], the results indicate that as the sample’s viscosity increases, its ability to maintain structural integrity and resist deformation also improves. These findings highlight the importance of a balanced approach in gel formulation, where viscosity is optimized to provide a desirable sensory experience without compromising the required physical properties. The study by Krop et al. [39] investigated the relationship of rheology and oral tribology with the sensory properties of hydrogels. The study found a strong correlation between the initial refractive properties of the hydrogels, bolus viscosity, and all texture attributes related to chewing, including the ones related to firmness, elasticity, chewability, and cohesiveness.

### 2.4. Online Survey

Differences in responses across age groups were tested by Pearson’s Chi-squared test of independence. The results are presented in Table 2 and indicate the calculated *p*-value was less than the significance level α = 0.05, which suggests that for all these hypotheses, we would reject the null hypothesis H_0_ in favor of the alternative hypothesis H_a_—indicating a significant link between age groups and the various aspects of edible gel preferences and concerns. 

The findings suggest that older adults had more prior experience with edible gels. Despite less experience, more than half (67.27%) of the younger participants were interested in trying edible gels, which was also observed in the middle-aged (40.08%) and older age groups (43.08%). These results suggest that Slovak respondents were generally interested in consuming new food products such as edible gels, and this interest was influenced by age, as hypothesized. However, the middle-aged group was the most skeptical, with up to 39.68% of respondents indicating that they would not be willing to try edible gels. This study also focused on the analysis of the preferred time of consumption of gels and thus it was found that young (72.73%) and older adults (72.31%) prefer to consume edible gels as a quick and convenient snack, while middle-aged adults (30.95%) are more inclined to consume them in the morning. The data imply that the preferred form of administration of edible gels varies between age groups. Most of the respondents from the young (68%), middle-aged (49.60%), and older adults (74.15%) categories preferred the tube form of administration. However, middle-aged adults (30.56%) showed relatively more interest in tablet or capsule formats such as xerogels compared to younger and older age groups. When examining the perceived risks associated with edible gel consumption, the link between the age category and existing concerns associated with edible gel consumption was again confirmed. However, it can be noted that in the younger and older generations, the largest number of respondents indicated that they did not perceive any concerns associated with edible gel consumption. Middle-aged respondents were mainly concerned about safety (48.02%) and health (32.94%) aspects.

**Figure 4:**
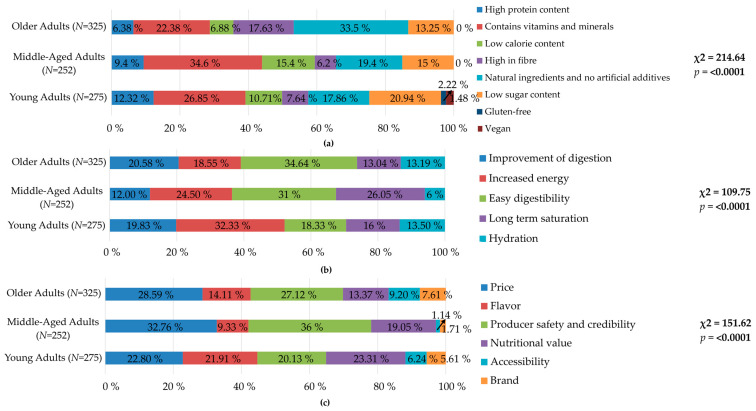
The results of an analysis studying (**a**) preferred properties or characteristics of edible gels, (**b**) expected effects or benefits, and (**c**) factors influencing the purchase decision.

In addition, this study investigated the preferred attributes and characteristics of edible gels, the expected effects or benefits associated with their consumption, and key factors influencing consumer purchase decisions. This approach aimed to provide a deeper understanding of the multifaceted aspects that determine consumers’ preferences and purchasing behavior in relation to edible gel products, and the results are presented in Figure 4.

The calculated *p*-value for all three asked questions was less than the significance level of α = 0.05. Therefore, the null hypothesis H_0_ was rejected, and the alternative hypothesis H_a_ was accepted. The youngest group of respondents showed the most diverse preferences, considering whether the products were gluten-free (2.22%) or vegan (1.48%), unlike the other age groups. For this age group, the decisive characteristics of edible gels were vitamin and mineral content, low sugar content, ingredients from natural sources, and the absence of artificial additives. Among the three age groups, the youngest placed the highest emphasis on high protein content. Notably, only 6.38% of the responses from the elderly group emphasized protein content as a key attribute. Considering that older adults have an increased need for protein due to age-related physiological changes, including sarcopenia and muscle loss, this finding is concerning [40]. Despite the increased need, the prevalence of inadequate protein intake is still alarmingly high in this demographic group [41,42]. A significant gap in awareness and understanding of older adults regarding their nutritional requirements is observed, particularly in protein intake. Ensuring adequate protein intake along with adequate energy intake is crucial for preventing malnutrition in the elderly. In addition, if the diet is inadequate, micronutrient deficiencies need to be addressed through supplementation [42]. Therefore, tailored educational interventions and public health initiatives are needed to inform and encourage this population to prioritize adequate protein consumption as part of their diet [43]. Increased awareness and knowledge can be crucial in promoting the consumption of functional foods [44]. Dietary fiber content was also highlighted as an important factor in this category of elderly people that would increase their consumption of products such as edible gels. Similarly, in a study by Vella et al. (2014), study participants frequently selected fiber and omega-3 fatty acids as desirable nutrients on functional food labels, suggesting that these bioactive components are particularly attractive to older adults and that low-sodium functional foods containing these components might be well-received by this demographic [44]. Similar findings were concluded in the study by the authors Szakos et al. (2020) [45], where for older adults, the increased vitamin, protein, and fiber content, as well as salt and sugar content were important factors for functional food preferences. Overall, respondents showed the greatest preference for nutritional claims related to vitamins and minerals. Protein, fiber, and sugar content were also important to respondents. Claims related to fat, energy, and salt content were moderately important. Lactose-free and gluten-free claims were the least important to respondents in forming preferences. 

For young adults, the expected health benefits and advantages of consuming edible gels included a preference for increased energy. The inclination toward energy-boosting products suggests that this age group is driven by an active lifestyle that may include a busy schedule or a demanding work environment that requires quick and convenient energy sources [46]. This preference may also be influenced by their familiarity with energy gels commonly offered to athletes, reflecting the perception of edible gels as products designed primarily for rapid energy delivery [47]. Benefits related to digestion were of moderate importance to young adults. The middle generation placed the greatest emphasis on easy digestibility (31%), long-term saturation (26.05%), and an increase in energy. The most important benefits for seniors were easy digestibility, improvement of digestion and hydration, and long-term saturation. Compared to all groups, this one placed less emphasis on increased energy. This indicates a divergence in dietary priorities among age groups, where younger individuals prioritize energy delivery, and older consumers are more interested in reducing disease risk [48]. 

Understanding the specific nutritional needs and preferences of different demographic segments, especially older adults, is essential for developing innovative food products that can effectively meet these needs while ensuring their acceptability and success in the marketplace [49,50]. Although a thorough and rigorous process is often applied in the development of food products designed to meet consumer preferences, the commercial success of these products is not guaranteed. A significant proportion of newly introduced food products will not achieve long-term market viability, despite extensive market research [51]. As a result, identifying the key factors that shape consumer purchasing decisions can be critical to the success of these products. Although various nutritional attributes were identified as important in consumers’ preferences concerning edible gel attributes, they were not decisive for respondents in the issues of factors determining their purchase decision for a given product. Nutritional value, price, flavor, and producer safety and credibility were similarly important considerations for young adults when considering the potential purchase of edible gels.

The results from this study indicate that there is a link between age categories and factors influencing the potential purchase of a new product such as an edible gel. Young adults had more balanced preferences across price, flavor, producer safety, and nutritional value. Middle-aged adults prioritize producer safety and credibility the most, as well as price. Older adults showed moderate concern for price and producer safety. Value for money and product safety seem to be key but were not the only factors considered in this category. This may be due to a lower pension compared to previous income [52] and increased health concerns. Accessibility and brand were generally less important factors across all age groups, whereas previous studies [53,54] suggested that brand was a dominant factor that influenced customers’ purchase intentions. Tabassum and Jabir (2020) [55] found that 44% of consumers are willing to pay up to a 9% premium for health and wellness foods reinforcing the notion that health awareness is a significant factor in purchasing decisions. This trend suggests that consumers in different age groups may be willing to invest more in products perceived as beneficial to their well-being.

## 3. Conclusions

This study analyzed consumer preferences regarding edible gels through a comprehensive approach that included an analysis of prototypes of BG- and AG-based gels targeted for seniors, along with an online survey that explored the preferences of three different age groups regarding edible gel consumption.

Statistically significant differences in the physical properties of the evaluated polysaccharide-based gels were found. Sensory evaluation involving eighty seniors revealed that the consumers preferred AG-based gels over BG-based gels, and the penalty analysis revealed that attributes such as intensity of flavor and bitter taste played a decisive role in older consumer perception. However, the intensity of the sweet taste was also an important factor affecting the overall liking of gels.

Textural properties had a significant impact on consumer preferences. A strong positive correlation was observed between the cohesiveness of the gels and their sensory properties, suggesting that hydrogels formulated from AG and BG should be less adhesive in order to be more attractive to consumers. Firmness was also identified as a key factor influencing consumer preferences for the appearance of edible gels, as the firmer the hydrogel, the less attractive it was to consumers in terms of appearance. Similar relationships were found for consistency. A negative correlation was also observed for viscosity, although it was not statistically significant.

Based on the results of the online survey, age-based matching strategies may prove more effective in examining factors influencing consumer response to edible gels, as relationships between age and respondents’ preferences were found. This highlights the importance of tailoring marketing strategies to reflect the age and sensory preferences of the target group. For younger consumers, edible gels could be designed as a quick snack to boost energy in convenient tube packaging focusing on natural ingredients, vitamin and mineral content, and high protein content. A strategy for the development of edible gels for middle-aged and younger consumers could focus on promoting balanced nutrition and satiety and their availability in both tube and tablet/capsule form (e.g., xerogels). Edible gel products tailored to older adult consumers could be designed to provide easy consumption, contain protein and fiber, and utilize a packaging format, such as a tube, that facilitates the usage. Promoting positive health and well-being outcomes for this demographic group should be a key objective.

The limitations of this study that should be considered in future research include the exclusive participation of seniors in consumer test, which limits the understanding of the preferences of other age groups. The smaller number of respondents may reduce the statistical power and generalizability of the findings. In addition, relying on self-reported data introduces potential biases and inaccuracies, while not accounting for demographic differences in the online survey limits the ability to generalize results across different population groups. Future research should also examine additional factors influencing preferences, such as gender.

## 4. Materials and Methods

### 4.1. Materials

BG (derived from *Pleurotus ostreatus*, 93% purity, M.W. 450 kDa) was supplied by Natures Ltd. (Trnava, Slovakia) as micronized particles. AG (sourced from *Larix sibirica*, 98.7% purity) was provided by the Favorskii Irkutsk Institute of Chemistry, Siberian Branch (Irkutsk, Russia) . Methocel 4A (MT) was procured from Sigma-Aldrich (St. Louis, MO, USA). The sweetener Palatinose (PT) was obtained from All media group s.r.o. (Bratislava, Slovakia). Chemicals such as sucrose (SC), citric acid (CA), and potassium sorbate (SK) used in the experiments were purchased locally and utilized without further modification. Double-distilled water was employed throughout this study, and flavorings were sourced from Aroco, s.r.o. (Prague, Czech Republic). The materials used in this study were food-grade and compliant with safety requirements.

### 4.2. Preparation of AG and BG Gels

The development of the edible gels evaluated in this study involved an iterative testing process. Different combinations of ingredients were investigated and formulations with target viscosities were selected for multiple sensory evaluations by a smaller group of evaluators (*N* = 15–20). Through this approach, the gel formulation described in this paper was determined. The procedure of AG:MT and BG:MT gels has been published in previous studies [24,56].

Samples were prepared in quantities of 100 g. The preparation of the AG hydrogel involved mixing 2 wt% AG in 98 mL of water, 10 wt% PT, 0.4 wt% CA, 0.03 wt% % SC, and 0.2 wt% SK. After heating the mixture to 82 °C, MT (2 wt %) was gradually added while stirring, with intermittent homogenization at 4000 rpm. Once the gel was cooled to a temperature of 45 °C, the aromatic component was incorporated in liquid form in quantities according to the manufacturer’s instructions.

BG gel was prepared by gelling the micronized 3 wt% BG in 97 mL of water for 48 h at 65 °C. Subsequently, other chemicals, such as 0.4 wt% CA, 0.03 wt% SC, and 0.2 wt% SK were added, the mixture was then stirred for 2 h at 82 °C with intermittent homogenization at 4000 rpm, and 1.6 wt% of MT was added. Once the MT was fully incorporated, the mixture was cooled to 45 °C, and then the flavoring was added in liquid form in quantities according to the manufacturer’s instructions.

**Figure 5:**
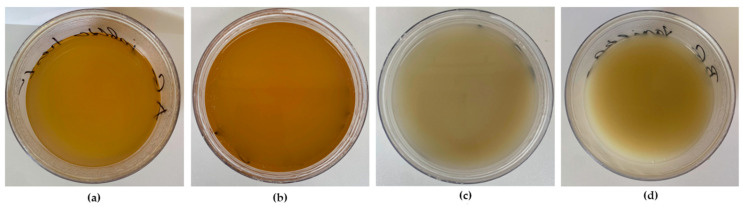
Prepared samples of edible gels: (**a**) arabinogalactan gel with coffee-biscuit flavor; (**b**) arabinogalactan gel with vanilla flavor; (**c**) beta-glucan with coffee-biscuit flavor; (**d**) beta-glucan gel with vanilla flavor.

Following this procedure, two variants of AG and BG gels were prepared, to which the following flavors were added: vanilla and coffee-biscuit. The flavor selection process involved pilot sensory evaluations of edible gel samples with various flavors, including apple, apple-crush, strawberry, raspberry, apricot, vanilla, coffee, kiwi, passion fruit, banana, latte, and coffee-biscuit, among others. Based on preliminary results from sensory evaluation sessions conducted with a smaller group of evaluators (*N* = 15–20) (data not shown), the flavors were chosen for the subsequent phase of the edible gel development project (APVV-20-0078) and for this study. The prepared samples are shown in Figure 5.

### 4.3. Viscosity Measurement

The viscosity of gels was measured using a Brookfield viscosimeter Model DV2T HA Brookfield Engineering Laboratories, Inc, Middleboro, MA, USA with a spindle T-B (92) at 20 °C and 200 min^−1^. All measurements were performed in triplicates. The temperature of the samples was monitored throughout the measurement process, maintaining a value of 22 ± 0.5 °C.

### 4.4. Texture Measurement

A TA.XT. plus texture analyzer (Stable Micro Systems, Surrey, UK), equipped with a cylindrical stainless-steel back-extrusion A/BE probe measuring 35 mm in diameter and a 5 kg load cell, was used for the analysis of gel textural properties. A 40 g sample was placed in a standard 40 mm diameter back extrusion container and subjected to compression at a speed of 1 mm/s. The penetration depth of the probe was set at 30 mm. Strength, consistency, cohesiveness, and work of cohesion were analyzed. The measurements were conducted in triplicates, and the temperature of the samples was 22 ± 0.5 °C.

### 4.5. Consumer Test

A total of 80 evaluators from Slovakia, comprising a group of seniors, participated in the consumer test. Their demographic information is illustrated in Table 3. The sensory questionnaire consisted of two sections. The first part focused on the evaluation of appearance, aroma, taste, aftertaste, and overall liking, using a nine-point hedonic scale [57] ranging from one—”dislike extremely”—to nine—”like extremely”. Subsequently, in the second part, the evaluators used a three-point JAR scale (one—”too little”, two—”just about right”, and three—”too much”) to rate attributes such as fluidity, color, intensity of sweet taste, bitter taste, and flavor.

The evaluation was carried out in September 2023 in the sensory laboratory of the Faculty of Biotechnology and Food Science of the Slovak University of Agriculture in Nitra (constituted according to the ISO 8589:2007 standards [58]). The sensory evaluation was carried out at an ambient temperature of 21–23 °C and a relative humidity of 65%. The lighting in the booths was standardized to 6500K, which simulated daylight conditions to avoid any color and visual distortion. Consumers were trained and familiarized with the methodology. The first session served as an introduction to the evaluation process. The second session involved the actual sensory evaluation, where participants evaluated presented samples. Samples were presented using a randomized complete block design (RCBD). The evaluators were administered 10–15 mL samples labeled with a three-digit code. Participants provided informed consent that outlined potential risks and their ability to withdraw from the evaluation at any time. Evaluator responses were collected anonymously, preventing the identification of individual participants.

### 4.6. Online Survey

This study collected quantitative data through an online survey conducted in Slovakia from January to May 2024. A total of 852 participants took part in the online survey. Respondents were divided into three categories based on age: young adults (18–39 years), middle-aged adults (40–59 years), and older adults (60+ years). Of the total number of participants, 275 (male: 127; female: 148) belonged to the young adult category, 252 (male: 121; female: 131) belonged to the older adult category, and 325 (male: 159; female 166) belonged to the older adult category. The questionnaire comprised questions on age and gender, and the survey questionnaire was designed to investigate hypotheses (see Table 4) assessing the relationship between the age groups and the various aspects of edible gel preferences and concerns. The questionnaire also consisted of multiple-choice questions that addressed consumers’ preferences regarding the desired characteristics and properties of edible gels, the expected benefits of such products, and the factors that would influence their potential to purchase such a product if it existed on the market.

### 4.7. Statistical Analysis

Descriptive statistics were used to summarize and interpret the results. An ANOVA with Duncan’s test and REGWQ were used to identify significant differences between the analyzed samples. A linear discriminant analysis (LDA) was used to improve classification. LDA is a supervised learning technique used to find linear combinations of features that best separate or discriminate between predefined classes [59]. A penalty analysis was used to evaluate the data obtained using the JAR scale, and the limit was set at 20% [60]. For all statistical analyses, the significance level was set at *p* ≤ 0.05. A Chi-square test of independence was used to test the existence of statistically significant relationships between age groups and preferences in accordance with the formulated hypotheses. A dependency was considered statistically significant if the *p*-value was below the α = 0.05 significance level. Statistical analyses were performed using Microsoft Excel (version 16.0.17928.20114) and XLSTAT (Addinsoft, 2024, New York, NY, USA). Pearson correlation (R Core Team, 2021) was used to assess the correlation between physical parameters and sensory properties.

## Figures and Tables

**Table 1:** ANOVA analysis—Ducan and REGWQ tests for viscosity and texture analysis of edible gel samples.

Sample	Viscosity (Pa·s), SD	Firmness (g), SD	Consistency (g.sec), SD	Cohesiveness (g), SD	Work of Cohesion (g.sec), SD
BGCC	1.37 ± 0.01 ^a^	15.33 ± 0.23 ^a^	143.03 ± 1.17 ^a^	−13.30 ± 0.36 ^d^	−51.12 ± 2.50 ^d^
BGV	1.27 ± 0.01 ^b^	14.18 ± 0.12 ^b^	136.98 ± 1.66 ^b^	−12.72 ± 0.10 ^c^	−43.61 ± 1.26 ^c^
AGCC	1.12 ± 0.01 ^c^	12.04 ± 0.13 ^c^	130.26 ± 0.91 ^c^	−11.37 ± 0.11 ^b^	−34.28 ± 1.36 ^b^
AGV	0.68 ± 0.01 ^d^	10.47 ± 0.03 ^d^	126.18 ± 0.30 ^d^	−10.80 ± 0.25 ^a^	−29.33 ± 0.65 ^a^

Notes: a, b, c, d = groups within a column with different superscripts differ significantly at *p* ≤ 0.05. BGCC—beta-glucan gel with coffee-biscuit flavor; BGV—beta-glucan gel with vanilla flavor; AGCC—arabinogalactan gel with coffee-biscuit flavor; AGV—arabinogalactan gel with vanilla flavor, SD—standard deviation.

**Table 2:** Results of the Chi-square test.

Classification	Question	Responses	Young Adults (*N* = 275)		Middle-Aged Adults (*N* = 252)		Older Adults (*N* = 325)		χ^2^	*p*-Value
			*N*	*%*	*N*	*%*	*N*	*%*		
**Familiarity and Willingness to Try Edible Gels**	Have you encountered a novel type of food product, such as edible gels?	No, but I would be curious to try it	185	67.27	101	40.08	140	43.08	164.91	<0.0001
		I’m not sure if I would have the courage	45	16.36	100	39.68	25	7.69		
		Yes, I’ve tried it	45	16.36	51	20.24	160	49.23		
**Preferred** **Consumption Timing**	When would be your preferred time to consume edible gels?	Whenever I feel the need for a quick and convenient snack	200	72.73	99	39.29	235	72.31	123.96	<0.0001
		In the morning	32	11.64	78	30.95	41	12.62		
		When doing sports	10	3.64	0	0.00	5	1.54		
		I don’t know	16	5.82	24	9.52	9	2.77		
		In the evening	10	3.64	28	11.11	23	7.08		
		When travelling	4	1.45	0	0.00	2	0.62		
		In case of emergency only	3	1.09	23	9.13	10	3.08		
**Preferred** **Packaging and Form**	Which form of administration do you think is the most appropriate for consuming edible gels?	Tube packaging	195	70.91	125	49.60	244	75.08	109.50	<0.0001
		Tablets or capsules	19	6.91	77	30.56	37	11.38		
		Ecological choice	11	4.00	0	0.00	0	0.00		
		Powdered form	15	5.45	2	0.79	6	1.85		
		In the form of porridge	35	12.73	48	19,05	38	11.69		
**Concerns about Consuming** **Edible Gels**	Would you have concerns about consuming edible gels?	No, I wouldn’t have	143	52.00	48	19.05	157	48.31	131.64	<0.0001
		Yes, safety concerns	23	8.36	121	48.02	69	21.23		
		Yes, health concerns	109	39.64	83	32.94	99	30.46		

**Table 3:** Demographics of evaluators participating in this study.

Category	*N*	%
Gender		
Male	24	70
Female	56	30
Age		
65–69	12	15
70–74	36	45
75 and over	32	40
Work		
Retired	79	99
Part-time employee	0	0
Full-time employee	0	0
Other	1	1
Residential Arrangement		
Living with a spouse	55	55
Living with family	10	10
Living alone	35	35
Living in a retirement home	0	0
Other	0	0
Chronic diseases		
Yes	47	59
No	33	41
Daily nutritional supplementation		
Yes	41	51
No	39	49
Dentures		
Yes	36	45
No	44	50
Chewing Difficulties		
Yes	28	35
No	52	65
Food Avoidance Patterns		
Yes	32	40
No	48	60

**Table 4:** Tested hypothesis on the relationship between age groups and consumer preferences.

Classification	H_0_	H_a_
Familiarity and Willingness to Try	There is no association between age group and willingness to try edible gels.	There is an association between age group and willingness to try edible gels.
Preferred Consumption Timing	There is no association between age group and preferred consumption timing of edible gels.	There is an association between age group and preferred consumption timing of edible gels.
Preferred Form of Edible Gels	There is no association between age group and preferred form of edible gels.	There is an association between age group and preferred form of edible gels.
Concerns about Consuming Edible Gels	There is no association between age group and concerns about consuming edible gels.	There is an association between age group and concerns about consuming edible gels.
Preferred Attributes or Characteristics	There is no association between age group and preferred properties of edible gels.	There is an association between age group and preferred properties of edible gels.
Expected Effects or Benefits	There is no association between age group and the expected benefits of edible gels.	There is an association between age group and the expected benefits of edible gels.
Purchase Decision Factors	There is no association between age group and factors influencing purchase decisions.	There is an association between age group and factors influencing purchase decisions.

## Data Availability

The raw/processed data required to reproduce these findings are available from the authors upon request.
